# Iodido[5-methyl-1*H*-benzimidazole-2(3*H*)-thione-κ*S*]bis­(triphenyl­phosphane-κ*P*)copper(I) methanol monosolvate

**DOI:** 10.1107/S1600536812039165

**Published:** 2012-09-26

**Authors:** Yu-Han Jiang, Qi-Ming Qiu, Rui-Xia Jiang, Xu Huang, Qiong-Hua Jin

**Affiliations:** a Department of Chemistry, Capital Normal University, Beijing 100048, People’s Republic of China; b Qingdao Hygain Chemical (Group) Co. Ltd, Qingdao 266044, People’s Republic of China

## Abstract

In the title compound, [CuI(C_8_H_8_N_2_S)(C_18_H_15_P)_2_]·CH_3_OH, the coordination environment around the Cu^I^ atom is distorted tetra­hedral, defined by two P atoms of two triphenyl­phosphane ligands, one S atom of a 5-methyl-1*H*-benzimidazole-2(3*H*)-thione ligand and one I atom. The complex mol­ecules and the methanol solvent mol­ecules are connected *via* N—H⋯O and O—H⋯I hydrogen bonds, forming a chain along [010]. An intra­molecular N—H⋯I hydrogen bond is also observed.

## Related literature

For the structures and properties of transition metal complexes with phosphanes, see: Baxter *et al.* (1994 ▶); Kitagawa *et al.* (1995 ▶); Lewis *et al.* (1996 ▶). For complexes with a 2-mercapto-5-methyl­benzimidazole ligand, see: Ozturk *et al.* (2009 ▶); Schneider *et al.* (2008 ▶). For related structures, see: Aslanidis *et al.* (1993 ▶); Li *et al.* (2004 ▶); Lobana *et al.* (2005 ▶).
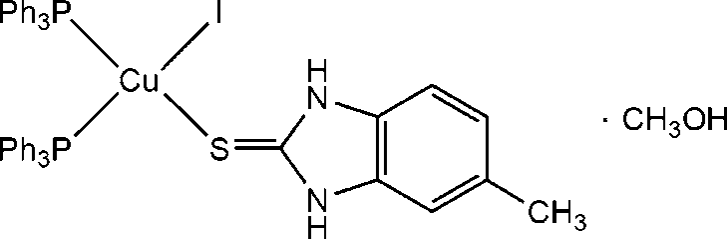


## Experimental

### 

#### Crystal data

[CuI(C_8_H_8_N_2_S)(C_18_H_15_P)_2_]·CH_4_O
*M* *_r_* = 911.27
Monoclinic, 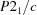

*a* = 15.6081 (11) Å
*b* = 10.5938 (8) Å
*c* = 25.976 (2) Å
β = 96.919 (1)°
*V* = 4263.8 (5) Å^3^
*Z* = 4
Mo *K*α radiation
μ = 1.40 mm^−1^
*T* = 298 K
0.43 × 0.38 × 0.35 mm

#### Data collection

Bruker APEX CCD diffractometer
Absorption correction: multi-scan (*SADABS*; Bruker, 2001 ▶) *T*_min_ = 0.585, *T*_max_ = 0.641
20808 measured reflections
7504 independent reflections
5465 reflections with *I* > 2σ(*I*)
*R*_int_ = 0.037

#### Refinement

*R*[*F* ^2^ > 2σ(*F* ^2^)] = 0.039
*wR*(*F* ^2^) = 0.088
*S* = 1.09
7504 reflections
478 parameters
H-atom parameters constrained
Δρ_max_ = 0.75 e Å^−3^
Δρ_min_ = −0.54 e Å^−3^

### 

Data collection: *SMART* (Bruker, 2007 ▶); cell refinement: *SAINT-Plus* (Bruker, 2007 ▶); data reduction: *SAINT-Plus*; program(s) used to solve structure: *SHELXS97* (Sheldrick, 2008 ▶); program(s) used to refine structure: *SHELXL97* (Sheldrick, 2008 ▶); molecular graphics: *SHELXTL* (Sheldrick, 2008 ▶); software used to prepare material for publication: *SHELXTL*.

## Supplementary Material

Crystal structure: contains datablock(s) global, I. DOI: 10.1107/S1600536812039165/hy2586sup1.cif

Structure factors: contains datablock(s) I. DOI: 10.1107/S1600536812039165/hy2586Isup2.hkl

Additional supplementary materials:  crystallographic information; 3D view; checkCIF report

## Figures and Tables

**Table 1:** Hydrogen-bond geometry (Å, °)

*D*—H⋯*A*	*D*—H	H⋯*A*	*D*⋯*A*	*D*—H⋯*A*
N1—H1⋯O1^i^	0.86	1.97	2.755 (7)	152
N2—H2⋯I1	0.86	2.80	3.539 (3)	145
O1—H1*A*⋯I1	0.82	2.67	3.469 (5)	164

Symmetry code: (i) 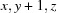.

**Fig. 1.**
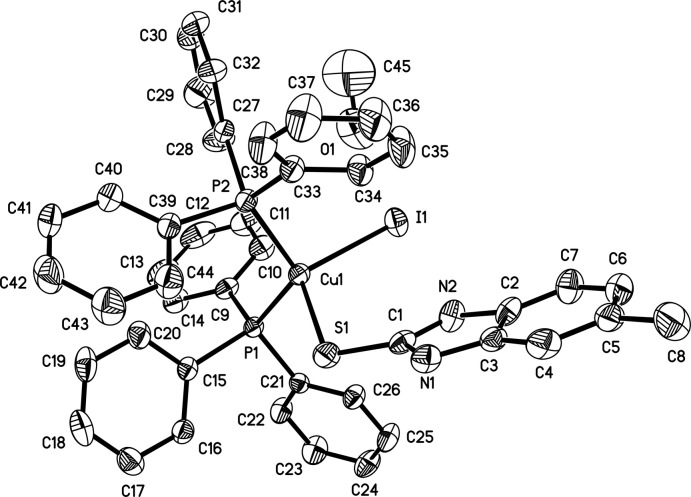
The molecular structure of the title compound. Displacement ellipsoids are drawn at the 50% probability level. H atoms have been omitted for clarity.

## supplementary crystallographic information

### Comment

Various transition metal complexes with bridging phosphanes or functionalized phosphanes have drawn much attention in recent years for their special structures, novel reactivity performances, catalytic properties and luminescence (Baxter *et al.*, 1994; Kitagawa *et al.*, 1995; Lewis *et al.*, 1996). The 2-mercapto-5-methylbenzimidazole (MMBI) ligand, with an -SH group and two potential coordination N atoms, is excellent in building supramolecular structures (Ozturk *et al.*, 2009; Schneider *et al.*, 2008). However, to our best knowledge, Cu(I) complexes with the MMBI ligand have not been reported. In this paper, one Cu(I) complex with PPh_3_ and MMBI is reported.

In the title compound, MMBI acts as a neutral, monodentate ligand with the S atom as a coordination atom. Other sites of the coordination tetrahedron are occupied by two P atoms from two PPh_3_ ligands and an iodide anion (Fig. 1). The Cu—S, Cu—P and Cu—I bond lengths agree with those in [CuI(H_2_itsc)(Ph_3_P)_2_] (H_2_itsc = isatin-3-thiosemicarbazones) (Lobana *et al.*, 2005) and [CuI(C_4_H_6_N_2_S)(C_18_H_15_P)_2_] (Li *et al.*, 2004). Similarly, in the title complex, angles around the Cu atom ranging from 102.31 (4) to 122.72 (4)° are close to those in [CuI(PPh_3_)_2_(pymtH)] (pymtH = pyrimidine-2-thione) (Aslanidis *et al.*, 1993). The complex molecules and the solvent methanol molecules are connected *via* N—H···O and O—H···I hydrogen bonds (Table 1), forming a chain along [0 1 0]. An intramolecular N—H···I hydrogen bond is also observed.

### Experimental

A mixture of CuI (0.2 mmol) and 2-mercapto-5-methylbenzimidazole (0.2 mmol) in MeOH and CH_2_Cl_2_ (10 ml, v/v = 1:1) was stirred for 2 h. PPh_3_ (0.2 mmol) was added to the mixture, which was stirred for another 4 h. The insoluble residues were removed by filtration, and filtrate was evaporated slowly at room temperature for a week to yield colorless crystalline products.

### Refinement

H atoms were positioned geometrically and refined as riding atoms, with C—H = 0.93 (aromatic) and 0.96 (CH_3_), N—H = 0.86 and O—H = 0.82 Å and with *U*_iso_(H) = 1.2(1.5 for methyl and hydroxyl)*U*_eq_(C,N,O).

### Crystal data

**Table:**

[CuI(C_8_H_8_N_2_S)(C_18_H_15_P)_2_]·CH_4_O	*F*(000) = 1848
*M**_r_* = 911.27	*D*_x_ = 1.420 Mg m^−^^3^
Monoclinic, *P*2_1_/*c*	Mo *K*α radiation, λ = 0.71073 Å
Hall symbol: -P 2ybc	Cell parameters from 8084 reflections
*a* = 15.6081 (11) Å	θ = 2.3–26.4°
*b* = 10.5938 (8) Å	µ = 1.40 mm^−^^1^
*c* = 25.976 (2) Å	*T* = 298 K
β = 96.919 (1)°	Block, colorless
*V* = 4263.8 (5) Å^3^	0.43 × 0.38 × 0.35 mm
*Z* = 4	

### Data collection

**Table:**

Bruker APEX CCD diffractometer	7504 independent reflections
Radiation source: fine-focus sealed tube	5465 reflections with *I* > 2σ(*I*)
Graphite monochromator	*R*_int_ = 0.037
φ and ω scans	θ_max_ = 25.0°, θ_min_ = 2.3°
Absorption correction: multi-scan (*SADABS*; Bruker, 2001)	*h* = −18→17
*T*_min_ = 0.585, *T*_max_ = 0.641	*k* = −12→11
20808 measured reflections	*l* = −24→30

### Refinement

**Table:**

Refinement on *F*^2^	Primary atom site location: structure-invariant direct methods
Least-squares matrix: full	Secondary atom site location: difference Fourier map
*R*[*F*^2^ > 2σ(*F*^2^)] = 0.039	Hydrogen site location: inferred from neighbouring sites
*wR*(*F*^2^) = 0.088	H-atom parameters constrained
*S* = 1.09	*w* = 1/[σ^2^(*F*_o_^2^) + (0.0172*P*)^2^ + 5.6518*P*] where *P* = (*F*_o_^2^ + 2*F*_c_^2^)/3
7504 reflections	(Δ/σ)_max_ = 0.001
478 parameters	Δρ_max_ = 0.75 e Å^−^^3^
0 restraints	Δρ_min_ = −0.54 e Å^−^^3^

### Special details

**Table:**

Geometry. All e.s.d.'s (except the e.s.d. in the dihedral angle between two l.s. planes) are estimated using the full covariance matrix. The cell e.s.d.'s are taken into account individually in the estimation of e.s.d.'s in distances, angles and torsion angles; correlations between e.s.d.'s in cell parameters are only used when they are defined by crystal symmetry. An approximate (isotropic) treatment of cell e.s.d.'s is used for estimating e.s.d.'s involving l.s. planes.
Refinement. Refinement of *F*^2^ against ALL reflections. The weighted *R*-factor *wR* and goodness of fit *S* are based on *F*^2^, conventional *R*-factors *R* are based on *F*, with *F* set to zero for negative *F*^2^. The threshold expression of *F*^2^ > σ(*F*^2^) is used only for calculating *R*-factors(gt) *etc*. and is not relevant to the choice of reflections for refinement. *R*-factors based on *F*^2^ are statistically about twice as large as those based on *F*, and *R*- factors based on ALL data will be even larger.

### Fractional atomic coordinates and isotropic or equivalent isotropic displacement parameters (Å^2^)

**Table:**

	*x*	*y*	*z*	*U*_iso_*/*U*_eq_	
Cu1	0.29356 (3)	0.60884 (4)	0.391446 (18)	0.03756 (13)	
I1	0.426976 (18)	0.44881 (3)	0.414919 (13)	0.05740 (11)	
N1	0.4673 (2)	0.9549 (3)	0.43122 (14)	0.0537 (9)	
H1	0.4405	1.0245	0.4233	0.064*	
N2	0.4928 (2)	0.7598 (3)	0.45022 (14)	0.0538 (10)	
H2	0.4850	0.6814	0.4569	0.065*	
O1	0.3820 (4)	0.1429 (4)	0.3723 (3)	0.134 (2)	
H1A	0.4018	0.2133	0.3794	0.201*	
P1	0.18454 (6)	0.53857 (10)	0.43498 (4)	0.0349 (2)	
P2	0.27571 (7)	0.64671 (10)	0.30391 (4)	0.0392 (3)	
S1	0.32156 (7)	0.81432 (10)	0.42639 (5)	0.0535 (3)	
C1	0.4289 (3)	0.8418 (4)	0.43596 (15)	0.0453 (10)	
C2	0.5732 (3)	0.8173 (4)	0.45291 (16)	0.0499 (11)	
C3	0.5561 (3)	0.9423 (4)	0.44091 (16)	0.0481 (10)	
C4	0.6229 (3)	1.0295 (4)	0.44001 (19)	0.0647 (14)	
H4	0.6114	1.1140	0.4323	0.078*	
C5	0.7071 (3)	0.9861 (5)	0.4510 (2)	0.0641 (14)	
C6	0.7220 (3)	0.8599 (5)	0.46243 (19)	0.0668 (14)	
H6	0.7786	0.8322	0.4698	0.080*	
C7	0.6563 (3)	0.7739 (5)	0.46341 (19)	0.0663 (14)	
H7	0.6678	0.6892	0.4709	0.080*	
C8	0.7824 (3)	1.0764 (5)	0.4514 (3)	0.100 (2)	
H8A	0.8228	1.0612	0.4817	0.151*	
H8B	0.7618	1.1617	0.4521	0.151*	
H8C	0.8103	1.0636	0.4209	0.151*	
C9	0.1375 (3)	0.3813 (4)	0.42121 (15)	0.0431 (10)	
C10	0.1941 (3)	0.2831 (4)	0.41679 (17)	0.0565 (12)	
H10	0.2530	0.2991	0.4187	0.068*	
C11	0.1638 (4)	0.1607 (5)	0.4095 (2)	0.0775 (16)	
H11	0.2026	0.0946	0.4075	0.093*	
C12	0.0777 (5)	0.1367 (6)	0.4053 (2)	0.0850 (19)	
H12	0.0577	0.0545	0.3999	0.102*	
C13	0.0205 (4)	0.2325 (6)	0.4090 (2)	0.0826 (18)	
H13	−0.0384	0.2154	0.4062	0.099*	
C14	0.0498 (3)	0.3555 (5)	0.41675 (17)	0.0596 (13)	
H14	0.0106	0.4208	0.4190	0.072*	
C15	0.0922 (2)	0.6456 (4)	0.42431 (15)	0.0376 (9)	
C16	0.0621 (3)	0.7184 (4)	0.46264 (17)	0.0478 (11)	
H16	0.0869	0.7103	0.4969	0.057*	
C17	−0.0048 (3)	0.8032 (4)	0.4501 (2)	0.0631 (13)	
H17	−0.0248	0.8515	0.4760	0.076*	
C18	−0.0417 (3)	0.8166 (5)	0.3999 (2)	0.0678 (15)	
H18	−0.0863	0.8741	0.3918	0.081*	
C19	−0.0132 (3)	0.7459 (5)	0.3618 (2)	0.0672 (15)	
H19	−0.0388	0.7545	0.3277	0.081*	
C20	0.0537 (3)	0.6610 (5)	0.37340 (18)	0.0578 (12)	
H20	0.0732	0.6137	0.3470	0.069*	
C21	0.2100 (2)	0.5320 (3)	0.50569 (14)	0.0357 (9)	
C22	0.1545 (3)	0.4788 (4)	0.53712 (16)	0.0530 (12)	
H22	0.1025	0.4445	0.5223	0.064*	
C23	0.1752 (3)	0.4759 (5)	0.59040 (18)	0.0652 (14)	
H23	0.1369	0.4409	0.6112	0.078*	
C24	0.2523 (3)	0.5247 (5)	0.61249 (17)	0.0637 (13)	
H24	0.2663	0.5231	0.6483	0.076*	
C25	0.3087 (3)	0.5759 (5)	0.58182 (17)	0.0614 (13)	
H25	0.3615	0.6076	0.5968	0.074*	
C26	0.2874 (3)	0.5804 (4)	0.52853 (16)	0.0468 (11)	
H26	0.3256	0.6164	0.5079	0.056*	
C27	0.2603 (2)	0.5071 (4)	0.26301 (15)	0.0449 (10)	
C28	0.2276 (3)	0.4022 (5)	0.2840 (2)	0.0696 (14)	
H28	0.2157	0.4035	0.3182	0.083*	
C29	0.2119 (4)	0.2933 (6)	0.2545 (3)	0.098 (2)	
H29	0.1882	0.2230	0.2688	0.117*	
C30	0.2309 (4)	0.2887 (7)	0.2047 (3)	0.094 (2)	
H30	0.2210	0.2154	0.1853	0.113*	
C31	0.2642 (4)	0.3912 (7)	0.1840 (2)	0.0859 (19)	
H31	0.2773	0.3882	0.1500	0.103*	
C32	0.2792 (3)	0.5009 (5)	0.21231 (18)	0.0669 (14)	
H32	0.3022	0.5710	0.1973	0.080*	
C33	0.3667 (3)	0.7297 (4)	0.28087 (16)	0.0481 (11)	
C34	0.4472 (3)	0.7145 (5)	0.30815 (18)	0.0585 (13)	
H34	0.4540	0.6615	0.3369	0.070*	
C35	0.5177 (3)	0.7767 (6)	0.2934 (2)	0.0873 (19)	
H35	0.5714	0.7668	0.3127	0.105*	
C36	0.5095 (4)	0.8528 (6)	0.2506 (2)	0.093 (2)	
H36	0.5576	0.8928	0.2403	0.111*	
C37	0.4308 (4)	0.8694 (6)	0.2234 (2)	0.101 (2)	
H37	0.4248	0.9223	0.1946	0.122*	
C38	0.3591 (3)	0.8083 (6)	0.2381 (2)	0.0815 (18)	
H38	0.3054	0.8203	0.2190	0.098*	
C39	0.1847 (3)	0.7474 (4)	0.27877 (15)	0.0443 (10)	
C40	0.1187 (3)	0.7103 (5)	0.24166 (18)	0.0624 (13)	
H40	0.1197	0.6300	0.2273	0.075*	
C41	0.0506 (3)	0.7915 (6)	0.2253 (2)	0.0832 (17)	
H41	0.0064	0.7650	0.2005	0.100*	
C42	0.0491 (4)	0.9100 (6)	0.2459 (2)	0.0891 (19)	
H42	0.0045	0.9651	0.2342	0.107*	
C43	0.1126 (4)	0.9482 (5)	0.2836 (2)	0.0821 (16)	
H43	0.1105	1.0283	0.2981	0.099*	
C44	0.1801 (3)	0.8674 (5)	0.3002 (2)	0.0683 (14)	
H44	0.2229	0.8936	0.3261	0.082*	
C45	0.3990 (8)	0.1101 (10)	0.3249 (4)	0.204 (5)	
H45A	0.4585	0.1275	0.3217	0.306*	
H45B	0.3628	0.1576	0.2994	0.306*	
H45C	0.3880	0.0216	0.3197	0.306*	

### Atomic displacement parameters (Å^2^)

**Table:**

	*U*^11^	*U*^22^	*U*^33^	*U*^12^	*U*^13^	*U*^23^
Cu1	0.0345 (3)	0.0433 (3)	0.0356 (3)	−0.0078 (2)	0.0072 (2)	−0.0013 (2)
I1	0.04338 (17)	0.04799 (18)	0.0800 (2)	−0.00010 (14)	0.00389 (15)	0.00189 (16)
N1	0.063 (2)	0.034 (2)	0.064 (2)	−0.0099 (18)	0.0073 (19)	−0.0053 (17)
N2	0.055 (2)	0.043 (2)	0.061 (2)	−0.0163 (19)	−0.0026 (19)	0.0114 (18)
O1	0.152 (5)	0.051 (3)	0.198 (6)	0.022 (3)	0.022 (5)	0.008 (3)
P1	0.0314 (5)	0.0405 (6)	0.0333 (6)	−0.0095 (4)	0.0057 (4)	−0.0015 (4)
P2	0.0362 (6)	0.0484 (6)	0.0332 (6)	−0.0073 (5)	0.0047 (5)	0.0012 (5)
S1	0.0521 (7)	0.0440 (6)	0.0647 (8)	−0.0101 (5)	0.0080 (6)	−0.0121 (5)
C1	0.055 (3)	0.042 (2)	0.039 (2)	−0.013 (2)	0.005 (2)	−0.0073 (19)
C2	0.057 (3)	0.046 (3)	0.043 (3)	−0.017 (2)	−0.004 (2)	0.001 (2)
C3	0.050 (3)	0.047 (3)	0.048 (3)	−0.011 (2)	0.008 (2)	−0.011 (2)
C4	0.076 (4)	0.037 (3)	0.083 (4)	−0.017 (2)	0.019 (3)	−0.013 (2)
C5	0.054 (3)	0.065 (3)	0.075 (4)	−0.018 (3)	0.015 (3)	−0.025 (3)
C6	0.056 (3)	0.070 (4)	0.073 (4)	−0.008 (3)	0.001 (3)	−0.009 (3)
C7	0.062 (3)	0.058 (3)	0.076 (4)	−0.008 (3)	−0.004 (3)	0.009 (3)
C8	0.069 (4)	0.076 (4)	0.161 (6)	−0.029 (3)	0.035 (4)	−0.045 (4)
C9	0.048 (3)	0.046 (3)	0.036 (2)	−0.018 (2)	0.0087 (19)	−0.0029 (19)
C10	0.061 (3)	0.048 (3)	0.060 (3)	−0.013 (2)	0.007 (2)	−0.002 (2)
C11	0.110 (5)	0.045 (3)	0.077 (4)	−0.008 (3)	0.011 (3)	−0.003 (3)
C12	0.128 (6)	0.067 (4)	0.062 (4)	−0.054 (4)	0.021 (4)	−0.011 (3)
C13	0.083 (4)	0.100 (5)	0.067 (4)	−0.058 (4)	0.020 (3)	−0.017 (3)
C14	0.050 (3)	0.071 (3)	0.059 (3)	−0.028 (2)	0.012 (2)	−0.010 (2)
C15	0.031 (2)	0.045 (2)	0.037 (2)	−0.0108 (17)	0.0059 (18)	0.0059 (18)
C16	0.042 (2)	0.054 (3)	0.046 (3)	−0.003 (2)	0.001 (2)	−0.003 (2)
C17	0.054 (3)	0.053 (3)	0.083 (4)	0.004 (2)	0.009 (3)	−0.004 (3)
C18	0.043 (3)	0.065 (3)	0.094 (4)	0.009 (2)	0.002 (3)	0.026 (3)
C19	0.044 (3)	0.097 (4)	0.059 (3)	0.002 (3)	−0.002 (2)	0.032 (3)
C20	0.039 (3)	0.086 (4)	0.049 (3)	−0.004 (2)	0.009 (2)	0.008 (2)
C21	0.034 (2)	0.035 (2)	0.038 (2)	−0.0019 (17)	0.0062 (17)	−0.0004 (17)
C22	0.046 (3)	0.073 (3)	0.040 (3)	−0.015 (2)	0.004 (2)	0.008 (2)
C23	0.062 (3)	0.089 (4)	0.046 (3)	−0.019 (3)	0.013 (2)	0.017 (3)
C24	0.076 (4)	0.079 (4)	0.035 (3)	−0.015 (3)	−0.001 (2)	0.008 (2)
C25	0.054 (3)	0.080 (4)	0.047 (3)	−0.018 (3)	−0.008 (2)	0.003 (2)
C26	0.045 (2)	0.056 (3)	0.039 (2)	−0.012 (2)	0.0033 (19)	0.003 (2)
C27	0.034 (2)	0.063 (3)	0.036 (2)	0.000 (2)	−0.0015 (18)	−0.006 (2)
C28	0.081 (4)	0.062 (3)	0.067 (3)	−0.020 (3)	0.016 (3)	−0.022 (3)
C29	0.106 (5)	0.074 (4)	0.112 (6)	−0.025 (4)	0.008 (4)	−0.032 (4)
C30	0.074 (4)	0.100 (5)	0.101 (6)	0.014 (4)	−0.021 (4)	−0.063 (4)
C31	0.074 (4)	0.123 (6)	0.057 (4)	0.024 (4)	−0.006 (3)	−0.037 (4)
C32	0.068 (3)	0.091 (4)	0.042 (3)	0.009 (3)	0.009 (2)	−0.007 (3)
C33	0.041 (3)	0.063 (3)	0.041 (3)	−0.011 (2)	0.007 (2)	0.005 (2)
C34	0.043 (3)	0.077 (3)	0.056 (3)	−0.011 (2)	0.010 (2)	0.015 (2)
C35	0.041 (3)	0.138 (6)	0.084 (4)	−0.023 (3)	0.010 (3)	0.029 (4)
C36	0.058 (4)	0.133 (6)	0.090 (4)	−0.038 (4)	0.021 (3)	0.029 (4)
C37	0.079 (4)	0.144 (6)	0.080 (4)	−0.045 (4)	0.009 (3)	0.051 (4)
C38	0.056 (3)	0.118 (5)	0.068 (4)	−0.024 (3)	−0.002 (3)	0.041 (3)
C39	0.040 (2)	0.054 (3)	0.039 (2)	−0.006 (2)	0.0040 (19)	0.005 (2)
C40	0.057 (3)	0.076 (3)	0.052 (3)	0.005 (3)	−0.003 (2)	−0.004 (3)
C41	0.064 (4)	0.118 (5)	0.061 (4)	0.022 (4)	−0.017 (3)	−0.001 (3)
C42	0.086 (4)	0.099 (5)	0.081 (4)	0.037 (4)	0.000 (3)	0.029 (4)
C43	0.089 (4)	0.056 (3)	0.099 (5)	0.011 (3)	0.001 (4)	0.011 (3)
C44	0.067 (3)	0.054 (3)	0.081 (4)	0.000 (3)	−0.007 (3)	0.003 (3)
C45	0.287 (15)	0.139 (9)	0.187 (12)	0.045 (9)	0.035 (11)	0.054 (8)

### Geometric parameters (Å, º)

**Table:**

Cu1—P1	2.2786 (10)	C19—C20	1.383 (6)
Cu1—P2	2.2925 (11)	C19—H19	0.9300
Cu1—S1	2.3786 (12)	C20—H20	0.9300
Cu1—I1	2.6974 (6)	C21—C26	1.379 (5)
N1—C1	1.352 (5)	C21—C22	1.380 (5)
N1—C3	1.386 (5)	C22—C23	1.383 (6)
N1—H1	0.8600	C22—H22	0.9300
N2—C1	1.341 (5)	C23—C24	1.370 (6)
N2—C2	1.389 (5)	C23—H23	0.9300
N2—H2	0.8600	C24—C25	1.368 (6)
O1—C45	1.334 (10)	C24—H24	0.9300
O1—H1A	0.8200	C25—C26	1.385 (6)
P1—C15	1.829 (4)	C25—H25	0.9300
P1—C21	1.833 (4)	C26—H26	0.9300
P1—C9	1.839 (4)	C27—C28	1.363 (6)
P2—C27	1.820 (4)	C27—C32	1.386 (6)
P2—C33	1.831 (4)	C28—C29	1.390 (7)
P2—C39	1.833 (4)	C28—H28	0.9300
S1—C1	1.689 (4)	C29—C30	1.361 (8)
C2—C7	1.373 (6)	C29—H29	0.9300
C2—C3	1.378 (6)	C30—C31	1.344 (8)
C3—C4	1.395 (6)	C30—H30	0.9300
C4—C5	1.389 (7)	C31—C32	1.380 (7)
C4—H4	0.9300	C31—H31	0.9300
C5—C6	1.383 (7)	C32—H32	0.9300
C5—C8	1.514 (6)	C33—C34	1.376 (6)
C6—C7	1.374 (6)	C33—C38	1.382 (6)
C6—H6	0.9300	C34—C35	1.376 (6)
C7—H7	0.9300	C34—H34	0.9300
C8—H8A	0.9600	C35—C36	1.367 (7)
C8—H8B	0.9600	C35—H35	0.9300
C8—H8C	0.9600	C36—C37	1.352 (7)
C9—C10	1.378 (6)	C36—H36	0.9300
C9—C14	1.387 (6)	C37—C38	1.385 (6)
C10—C11	1.385 (6)	C37—H37	0.9300
C10—H10	0.9300	C38—H38	0.9300
C11—C12	1.360 (8)	C39—C40	1.380 (6)
C11—H11	0.9300	C39—C44	1.393 (6)
C12—C13	1.362 (8)	C40—C41	1.392 (7)
C12—H12	0.9300	C40—H40	0.9300
C13—C14	1.389 (7)	C41—C42	1.367 (8)
C13—H13	0.9300	C41—H41	0.9300
C14—H14	0.9300	C42—C43	1.369 (8)
C15—C16	1.385 (5)	C42—H42	0.9300
C15—C20	1.394 (5)	C43—C44	1.384 (7)
C16—C17	1.387 (6)	C43—H43	0.9300
C16—H16	0.9300	C44—H44	0.9300
C17—C18	1.368 (7)	C45—H45A	0.9600
C17—H17	0.9300	C45—H45B	0.9600
C18—C19	1.358 (7)	C45—H45C	0.9600
C18—H18	0.9300		
			
P1—Cu1—P2	122.72 (4)	C18—C19—H19	119.9
P1—Cu1—S1	102.91 (4)	C20—C19—H19	119.9
P2—Cu1—S1	102.31 (4)	C19—C20—C15	120.7 (5)
P1—Cu1—I1	106.54 (3)	C19—C20—H20	119.7
P2—Cu1—I1	109.42 (3)	C15—C20—H20	119.7
S1—Cu1—I1	112.81 (3)	C26—C21—C22	118.5 (4)
C1—N1—C3	110.1 (4)	C26—C21—P1	119.3 (3)
C1—N1—H1	125.0	C22—C21—P1	122.2 (3)
C3—N1—H1	125.0	C21—C22—C23	120.9 (4)
C1—N2—C2	111.5 (3)	C21—C22—H22	119.6
C1—N2—H2	124.2	C23—C22—H22	119.6
C2—N2—H2	124.2	C24—C23—C22	119.9 (4)
C45—O1—H1A	109.5	C24—C23—H23	120.1
C15—P1—C21	104.32 (17)	C22—C23—H23	120.1
C15—P1—C9	104.08 (18)	C25—C24—C23	120.0 (4)
C21—P1—C9	101.16 (17)	C25—C24—H24	120.0
C15—P1—Cu1	110.15 (12)	C23—C24—H24	120.0
C21—P1—Cu1	115.16 (12)	C24—C25—C26	120.1 (4)
C9—P1—Cu1	120.27 (13)	C24—C25—H25	120.0
C27—P2—C33	104.65 (19)	C26—C25—H25	120.0
C27—P2—C39	102.86 (19)	C21—C26—C25	120.7 (4)
C33—P2—C39	101.65 (19)	C21—C26—H26	119.7
C27—P2—Cu1	115.37 (14)	C25—C26—H26	119.7
C33—P2—Cu1	113.70 (14)	C28—C27—C32	118.3 (4)
C39—P2—Cu1	116.85 (13)	C28—C27—P2	117.3 (3)
C1—S1—Cu1	110.32 (15)	C32—C27—P2	124.4 (4)
N2—C1—N1	106.0 (4)	C27—C28—C29	120.4 (5)
N2—C1—S1	128.4 (3)	C27—C28—H28	119.8
N1—C1—S1	125.6 (4)	C29—C28—H28	119.8
C7—C2—C3	121.3 (4)	C30—C29—C28	120.6 (6)
C7—C2—N2	133.6 (4)	C30—C29—H29	119.7
C3—C2—N2	105.2 (4)	C28—C29—H29	119.7
C2—C3—N1	107.2 (4)	C31—C30—C29	119.4 (6)
C2—C3—C4	121.0 (4)	C31—C30—H30	120.3
N1—C3—C4	131.8 (4)	C29—C30—H30	120.3
C5—C4—C3	117.9 (4)	C30—C31—C32	121.0 (6)
C5—C4—H4	121.0	C30—C31—H31	119.5
C3—C4—H4	121.0	C32—C31—H31	119.5
C6—C5—C4	119.6 (4)	C31—C32—C27	120.3 (5)
C6—C5—C8	120.0 (5)	C31—C32—H32	119.9
C4—C5—C8	120.5 (5)	C27—C32—H32	119.9
C7—C6—C5	122.7 (5)	C34—C33—C38	118.0 (4)
C7—C6—H6	118.7	C34—C33—P2	118.1 (3)
C5—C6—H6	118.7	C38—C33—P2	123.9 (3)
C2—C7—C6	117.5 (5)	C33—C34—C35	120.8 (4)
C2—C7—H7	121.2	C33—C34—H34	119.6
C6—C7—H7	121.2	C35—C34—H34	119.6
C5—C8—H8A	109.5	C36—C35—C34	120.6 (5)
C5—C8—H8B	109.5	C36—C35—H35	119.7
H8A—C8—H8B	109.5	C34—C35—H35	119.7
C5—C8—H8C	109.5	C37—C36—C35	119.4 (5)
H8A—C8—H8C	109.5	C37—C36—H36	120.3
H8B—C8—H8C	109.5	C35—C36—H36	120.3
C10—C9—C14	118.7 (4)	C36—C37—C38	120.6 (5)
C10—C9—P1	117.1 (3)	C36—C37—H37	119.7
C14—C9—P1	124.2 (4)	C38—C37—H37	119.7
C9—C10—C11	120.4 (5)	C33—C38—C37	120.6 (5)
C9—C10—H10	119.8	C33—C38—H38	119.7
C11—C10—H10	119.8	C37—C38—H38	119.7
C12—C11—C10	120.2 (5)	C40—C39—C44	117.9 (4)
C12—C11—H11	119.9	C40—C39—P2	124.4 (4)
C10—C11—H11	119.9	C44—C39—P2	117.6 (3)
C11—C12—C13	120.4 (5)	C39—C40—C41	121.0 (5)
C11—C12—H12	119.8	C39—C40—H40	119.5
C13—C12—H12	119.8	C41—C40—H40	119.5
C12—C13—C14	120.1 (5)	C42—C41—C40	119.8 (5)
C12—C13—H13	119.9	C42—C41—H41	120.1
C14—C13—H13	119.9	C40—C41—H41	120.1
C9—C14—C13	120.1 (5)	C41—C42—C43	120.4 (5)
C9—C14—H14	119.9	C41—C42—H42	119.8
C13—C14—H14	119.9	C43—C42—H42	119.8
C16—C15—C20	118.2 (4)	C42—C43—C44	119.9 (5)
C16—C15—P1	124.4 (3)	C42—C43—H43	120.1
C20—C15—P1	117.3 (3)	C44—C43—H43	120.1
C15—C16—C17	120.1 (4)	C43—C44—C39	120.9 (5)
C15—C16—H16	119.9	C43—C44—H44	119.5
C17—C16—H16	119.9	C39—C44—H44	119.5
C18—C17—C16	120.6 (5)	O1—C45—H45A	109.5
C18—C17—H17	119.7	O1—C45—H45B	109.5
C16—C17—H17	119.7	H45A—C45—H45B	109.5
C19—C18—C17	120.0 (5)	O1—C45—H45C	109.5
C19—C18—H18	120.0	H45A—C45—H45C	109.5
C17—C18—H18	120.0	H45B—C45—H45C	109.5
C18—C19—C20	120.3 (5)		
			
P2—Cu1—P1—C15	−55.75 (15)	C20—C15—C16—C17	0.4 (6)
S1—Cu1—P1—C15	58.28 (14)	P1—C15—C16—C17	175.6 (3)
I1—Cu1—P1—C15	177.16 (14)	C15—C16—C17—C18	−0.3 (7)
P2—Cu1—P1—C21	−173.36 (13)	C16—C17—C18—C19	0.4 (7)
S1—Cu1—P1—C21	−59.33 (14)	C17—C18—C19—C20	−0.6 (8)
I1—Cu1—P1—C21	59.55 (14)	C18—C19—C20—C15	0.8 (7)
P2—Cu1—P1—C9	65.20 (17)	C16—C15—C20—C19	−0.6 (6)
S1—Cu1—P1—C9	179.23 (16)	P1—C15—C20—C19	−176.2 (3)
I1—Cu1—P1—C9	−61.90 (16)	C15—P1—C21—C26	−114.3 (3)
P1—Cu1—P2—C27	−69.86 (15)	C9—P1—C21—C26	137.9 (3)
S1—Cu1—P2—C27	175.81 (14)	Cu1—P1—C21—C26	6.6 (4)
I1—Cu1—P2—C27	55.97 (15)	C15—P1—C21—C22	66.4 (4)
P1—Cu1—P2—C33	169.21 (16)	C9—P1—C21—C22	−41.5 (4)
S1—Cu1—P2—C33	54.88 (17)	Cu1—P1—C21—C22	−172.8 (3)
I1—Cu1—P2—C33	−64.96 (16)	C26—C21—C22—C23	1.0 (7)
P1—Cu1—P2—C39	51.22 (16)	P1—C21—C22—C23	−179.6 (4)
S1—Cu1—P2—C39	−63.11 (16)	C21—C22—C23—C24	−0.9 (7)
I1—Cu1—P2—C39	177.05 (15)	C22—C23—C24—C25	−0.2 (8)
P1—Cu1—S1—C1	139.24 (16)	C23—C24—C25—C26	1.2 (8)
P2—Cu1—S1—C1	−92.61 (16)	C22—C21—C26—C25	−0.1 (6)
I1—Cu1—S1—C1	24.83 (16)	P1—C21—C26—C25	−179.5 (3)
C2—N2—C1—N1	−2.3 (5)	C24—C25—C26—C21	−1.0 (7)
C2—N2—C1—S1	178.6 (3)	C33—P2—C27—C28	150.5 (4)
C3—N1—C1—N2	2.0 (5)	C39—P2—C27—C28	−103.6 (4)
C3—N1—C1—S1	−178.8 (3)	Cu1—P2—C27—C28	24.8 (4)
Cu1—S1—C1—N2	−34.4 (4)	C33—P2—C27—C32	−29.7 (4)
Cu1—S1—C1—N1	146.7 (3)	C39—P2—C27—C32	76.2 (4)
C1—N2—C2—C7	−177.0 (5)	Cu1—P2—C27—C32	−155.4 (3)
C1—N2—C2—C3	1.7 (5)	C32—C27—C28—C29	−1.6 (8)
C7—C2—C3—N1	178.5 (4)	P2—C27—C28—C29	178.2 (4)
N2—C2—C3—N1	−0.4 (5)	C27—C28—C29—C30	1.7 (9)
C7—C2—C3—C4	−1.3 (7)	C28—C29—C30—C31	−0.8 (10)
N2—C2—C3—C4	179.8 (4)	C29—C30—C31—C32	−0.1 (9)
C1—N1—C3—C2	−1.0 (5)	C30—C31—C32—C27	0.1 (8)
C1—N1—C3—C4	178.7 (4)	C28—C27—C32—C31	0.7 (7)
C2—C3—C4—C5	0.7 (7)	P2—C27—C32—C31	−179.1 (4)
N1—C3—C4—C5	−179.0 (4)	C27—P2—C33—C34	−99.1 (4)
C3—C4—C5—C6	−0.2 (7)	C39—P2—C33—C34	154.1 (4)
C3—C4—C5—C8	−179.1 (5)	Cu1—P2—C33—C34	27.7 (4)
C4—C5—C6—C7	0.1 (8)	C27—P2—C33—C38	81.9 (5)
C8—C5—C6—C7	179.0 (5)	C39—P2—C33—C38	−24.9 (5)
C3—C2—C7—C6	1.2 (7)	Cu1—P2—C33—C38	−151.4 (4)
N2—C2—C7—C6	179.7 (5)	C38—C33—C34—C35	0.6 (8)
C5—C6—C7—C2	−0.6 (8)	P2—C33—C34—C35	−178.5 (4)
C15—P1—C9—C10	168.4 (3)	C33—C34—C35—C36	−1.5 (9)
C21—P1—C9—C10	−83.6 (3)	C34—C35—C36—C37	1.7 (11)
Cu1—P1—C9—C10	44.5 (4)	C35—C36—C37—C38	−1.1 (11)
C15—P1—C9—C14	−13.8 (4)	C34—C33—C38—C37	0.0 (9)
C21—P1—C9—C14	94.3 (4)	P2—C33—C38—C37	179.1 (5)
Cu1—P1—C9—C14	−137.7 (3)	C36—C37—C38—C33	0.3 (11)
C14—C9—C10—C11	−1.8 (7)	C27—P2—C39—C40	6.5 (4)
P1—C9—C10—C11	176.1 (4)	C33—P2—C39—C40	114.6 (4)
C9—C10—C11—C12	1.7 (8)	Cu1—P2—C39—C40	−121.0 (4)
C10—C11—C12—C13	−0.9 (8)	C27—P2—C39—C44	−176.4 (4)
C11—C12—C13—C14	0.3 (9)	C33—P2—C39—C44	−68.2 (4)
C10—C9—C14—C13	1.2 (7)	Cu1—P2—C39—C44	56.1 (4)
P1—C9—C14—C13	−176.6 (4)	C44—C39—C40—C41	1.4 (7)
C12—C13—C14—C9	−0.5 (8)	P2—C39—C40—C41	178.5 (4)
C21—P1—C15—C16	9.9 (4)	C39—C40—C41—C42	0.5 (8)
C9—P1—C15—C16	115.6 (3)	C40—C41—C42—C43	−2.0 (9)
Cu1—P1—C15—C16	−114.2 (3)	C41—C42—C43—C44	1.5 (9)
C21—P1—C15—C20	−174.9 (3)	C42—C43—C44—C39	0.5 (8)
C9—P1—C15—C20	−69.2 (3)	C40—C39—C44—C43	−1.9 (7)
Cu1—P1—C15—C20	61.0 (3)	P2—C39—C44—C43	−179.2 (4)

### Hydrogen-bond geometry (Å, º)

**Table:**

*D*—H···*A*	*D*—H	H···*A*	*D*···*A*	*D*—H···*A*
N1—H1···O1^i^	0.86	1.97	2.755 (7)	152
N2—H2···I1	0.86	2.80	3.539 (3)	145
O1—H1*A*···I1	0.82	2.67	3.469 (5)	164

Symmetry code: (i) *x*, *y*+1, *z*.
